# The role of the MYL12A liquid-liquid phase separation in neutrophil improves the prognosis of acute respiratory distress syndrome: a multi-omics analysis

**DOI:** 10.3389/fimmu.2025.1662680

**Published:** 2025-10-29

**Authors:** Yufang Guo, Longjie Li, Yongjun Wang, Zexu Wang, Wei Qiu, Qian Li, Li Wang, Bing Wan

**Affiliations:** ^1^ Department of Respiratory and Critical Care Medicine, The Affiliated Jiangning Hospital of Nanjing Medical University, Nanjing, China; ^2^ Department of Pulmonary and Critical Care Medicine, Suining Central Hospital, Suining, Sichuan, China; ^3^ School of Biomedical Engineering and Informatics, Nanjing Medical University, Nanjing, China; ^4^ Department of Pathology, The Affiliated Jiangning Hospital of Nanjing Medical University, Nanjing, China; ^5^ Department of Anesthesiology, The Affiliated Jiangning Hospital of Nanjing Medical University, Nanjing, China

**Keywords:** liquid-liquid phase separation, acute respiratory distress syndrome, neutrophils, single-cell RNA sequencing, MYL12A

## Abstract

**Background:**

In acute respiratory distress syndrome (ARDS), neutrophils, as the primary effector immune cells, undergo profound transcriptional and phenotypic reprogramming in response to complex inflammatory stimuli, modulating signal transduction and immune responses. Liquid-liquid phase separation (LLPS) plays a pivotal role in transcriptional dynamics and signal transduction, critically influencing gene expression stability. However, the mechanistic and clinical implications of LLPS in ARDS progression remain elusive.

**Materials and methods:**

This study systematically characterized neutrophil LLPS in ARDS through integrated single-cell transcriptomes (GSE157789), proteomes (GSE32707/GSE76293), and clinical cohorts date. LLPS-associated genes (LCGs) were screened from the PhaSepDB 2.1 database and subsequently integrated with single-cell sequencing data from Gene Expression Omnibus (GEO) to quantify neutrophil LLPS scores and divide patient stratification into high and low LLPS groups for differential expression analysis of critical LCGs and associated pathways. Phase-separated droplets were then isolated from peripheral blood neutrophils of ARDS patients and N-formylmethionyl-leucyl-phenylalanine (fMLP) -stimulated neutrophils, followed by proteomic identification of droplet-associated proteins and candidate gene selection through GEO data analysis. The prognostic value of LLPS scores and candidate genes was subsequently validated in clinical cohorts, while the relationship between phase separation of candidate genes and cellular function was experimentally confirmed through immunofluorescence, Western blotting, and complementary functional assays.

**Results:**

Neutrophils in ARDS exhibit elevated LLPS scores (*p*<0.05), with differentially expressed LCGs enriched in RhoA/ROCK-mediated cell polarization and migration pathways. Multi-omics integration identified MYL12A as a core phase-separation regulator, whose protein levels in phase-separated droplets positively correlated with oxygenation index (r=0.9536, *p*=0.0119) but inversely with SOFA scores (r=-0.8896, *p*=0.0433). Patients with high LLPS scores demonstrated significantly improved overall survival (*p*=0.046), suggesting a protective role of LLPS in ARDS pathogenesis. Mechanistically, the chemoattractant fMLP triggers reversible MYL12A phase separation via phosphorylation at Ser19, thereby potentiating neutrophil migratory capacity.

**Conclusions:**

This study demonstrates that LLPS dynamically regulates neutrophil migration through MYL12A phosphorylation-dependent phase separation, exerting immunoprotective effect in ARDS. The LLPS status of MYL12A and its activity score may serve as ARDS prognostic biomarkers and offer a novel strategy for developing LLPS-targeted immunomodulatory therapies.

## 1 Introduction

Acute respiratory distress syndrome (ARDS)—a life-threatening condition characterized by refractory hypoxemia, reduced lung compliance, and diffuse alveolar damage—arises from either direct pulmonary insult (e.g., pneumonia, aspiration) or indirect systemic injuries (e.g., sepsis, major trauma) (1). Despite advances in lung-protective ventilation strategies (e.g., low tidal volume ventilation, prone ventilation) and precision fluid management, ARDS mortality remains alarmingly high (35-46%) (2, 3), underscoring an urgent need to decipher its molecular pathogenesis for targeted therapeutic development.

Neutrophils, constituting 50-70% of circulating leukocytes, serve as the core component of the innate immune system (4, 5). Neutrophils orchestrate dual immunological functions: they mediate inflammatory responses through the release of reactive oxygen species (ROS) (6), proteolytic enzymes, and neutrophil extracellular traps (NETs) (7), while concurrently executing pathogen clearance via phagocytosis and antimicrobial peptide secretion. During ARDS progression, neutrophils infiltrate the alveolar space via a CD11b/CD18 integrin-dependent transendothelial migration pathway (8). However, overactivation of neutrophils triggers a pathological cascade—including NADPH oxidase-mediated respiratory burst, mitochondrial ROS (mtROS) overproduction, and excessive elastase/matrix metalloproteinase (MMP) release—culminating in alveolar-capillary barrier disruption and pulmonary edema (9–12). This “double-edged sword” nature poses significant challenges for neutrophil-targeted interventions strategies.

Liquid-liquid phase separation (LLPS) represents a fundamental biophysical process wherein biomolecules undergo phase transition through multivalent weak interactions (e.g., π-π stacking, electrostatic forces) (13), forming Membrane-free organelles with liquid-like properties (e.g., stress granules, nucleoli) (14, 15). LLPS dysregulation has been implicated in neurodegenerative disorders and oncogenesis (16). Emerging research reveals that type II alveolar epithelial cells utilize LLPS-mediated production of SGs to counteract oxidative damage in respiratory diseases (17), while SARS-CoV-2 hijacks host phase separation machinery to facilitate viral assembly (18, 19). These findings position LLPS as a critical regulator of pulmonary inflammation.

Within the ARDS-specific ‘cytokine storm’ microenvironment, neutrophils undergo profound transcriptional reprogramming and phenotypic switching (e.g., from pro-inflammatory N1 phenotype to anti-inflammatory N2 phenotype) — a process demanding exquisite molecular control to balance inflammatory responses and tissue repair (20). A key component of the cell migration machinery is non-muscle myosin II, whose activity is regulated by phosphorylation of its regulatory light chain, MYL12A. As a pivotal effector downstream of signaling pathways like RhoA/ROCK, MYL12A directly translates upstream signals into mechanical force by modulating the interaction between myosin and the actin cytoskeleton. This activation is primarily achieved through phosphorylation at Serine 19, a modification directly catalyzed by Rho-associated kinase (ROCK), a key effector of the RhoA signaling pathway known to be triggered by chemoattractants. However, the role of biophysical processes like LLPS in regulating this critical signaling-to-mechanics interface in ARDS neutrophils remains unknown. However, systematic understanding of how neutrophil LLPS dynamically governs migration, activation, and effector functions remains elusive. The specific expression patterns of LLPS-associated genes (LCGs) across neutrophil subsets and their clinical implications are particularly undefined. Through integrated single-cell transcriptomics, proteomics, and functional validation, this study pioneers the discovery that MYL12A orchestrates neutrophil migration via LLPS, unveiling a novel target for precision immunotherapy in ARDS.

## 2 Material and methods

### 2.1 Study approval

This experiment was approved by the Ethics Committee of Jiangning Hospital Affiliated to Nanjing Medical University (ethics number: 2025-03-047-K01), Participation in this study was subject to written informed consent from each patient.

### 2.2 Subjects

Peripheral blood whole blood samples (aged 65–82 years) of patients with ARDS were obtained in the Department of Respiratory and Critical Care Medicine. In addition, patients admitted to the hospital due to pulmonary nodules were selected as the control group (aged 52–80 years). Control groups reporting any type of infection, inflammation, history of tumor radiotherapy and chemotherapy, diseases of the rheumatic system were excluded. Patients with ARDS excluded a history of malignant tumors, a history of tumor radiotherapy and chemotherapy, and diseases of the rheumatic system. A detailed information description of the enrolled ARDS patients was presented in **Table 1**.

**Table 1:** Clinical characteristics of 5 patients with ARDS.

Variables	Sample1	Sample2	Sample3	Sample4	Sample5
Gender	Male	Male	Female	Male	Male
Age (years)	65	80	74	79	72
FiO_2_, %	40	45	50	50	55
PaO_2_, mmHg	88	90	77	74	81
PaCO_2_, mmHg	49	44	43	51	48
PaO_2/_FiO_2_(mmHg)	220	200	154	148	147
PEEP, cm H_2_O	14	15	15	15	15
SaO_2_, %	97	96	98	94	99
SOFA score at inclusion	12	14	20	17	19
Cause of ARDS	CAP	Aspiration	Aspiration	CAP	CAP
Lactates, mmol/L	1.4	1.2	1.7	1.8	1.6

FiO_2_ Fraction of inspired oxygen; PO_2_ Oxygen partial pressure; PCO_2_ Partial pressure of carbon dioxide; PaO_2_/FiO_2_ the ratio of the partial pressure of arterial oxygen to the fraction of inspired oxygen; SOFA Sequential organ failure assessment score; SaO_2_ Arterial oxygen saturation; ARDS Acute respiratory distress syndrome; PaO_2_ Partial pressure of arterial oxygen; PaCO_2_ Partial pressure of arterial carbon dioxide; CAP Community-acquired pneumonia.

### 2.3 Isolation and culture of neutrophils from peripheral blood

Human peripheral blood neutrophil isolation kit (Solaibao, China) was used. An appropriate amount of peripheral venous blood was drawn into a vacuum blood collection tube containing lithium heparin with a sterile lancet, and the whole blood was carefully spread on top of the separating solution in a centrifuge tube, and two ring-shaped milky white layers were seen after centrifugation at 1000xg for 30min at room temperature, the upper layer was a monocyte layer, and the lower layer was a neutrophil layer, the lower layer of neutrophils was collected, the red blood cell lysate was added to eliminate the red blood cells, and the human primary peripheral blood neutrophils were obtained after centrifugation again, and the cell viability and cell purity were ≥ 90%. Neutrophils were seeded in RPMI-1640 medium containing 10% fetal bovine serum (Sigma, USA) and 1% penicillin/streptomycin (Sigma, USA) bispecific antibody in suspension at 37°C in an incubator containing 5% CO_2_.

### 2.4 Human neutrophil transwell migration assay

Isolated human peripheral blood neutrophils were identified for cell migration function in an 8 μm multimembrane well chamber (Nest, China). Cells were seeded into the upper chamber at a density of 10^5^ cells/mL at 100 μL/well. Neutrophils were treated with or without 1,6-hexanediol (1,6-HD) (Aladdin, China) and anti-MYL12A (Abcam, USA), migrated to the lower chamber for 30 min with or without N-formylmethionyl-leucyl-phenylalanine (fMLP) (Aladdin, China) stimulation, and cells migrated in the lower chamber were collected and quantified using an automated cell counter to calculate cell mobility.

### 2.5 Protein profiling of neutrophil phase separation droplets

In this study, the cytoplasmic phase separation components were extracted according to the method of Arnaud Hubstenberger et al. (21). Firstly, collected cell samples were treated with RNase inhibitors (Thermo Fisher Scientific, USA) to inhibit RNA degradation. Subsequently, ultrasonic discrushing was performed under ice bath conditions with a Sonic Disembriator 500 sonicator (Thermo Fisher Scientific, USA). After disruption, the nuclei and DNA components were removed by centrifugation, and the supernatant was transferred to a BD Biosciences FACSAria Fusion Flow Cytometer, where the phase separation coagulation was purified at 4°C. Ultimately, we obtained peripheral blood neutrophil proteomic data from 5 ARDS patients and 5 healthy volunteers (**Supplementary Table 1**), and neutrophil proteomic data from 5 fMLP-stimulated and 5 control groups (**Supplementary Table 2**).

### 2.6 Immunofluorescence assay of MYL12A and p-MYL12A expression in neutrophils

Cells were fixed with 4% paraformaldehyde (Beyotime, China), permeabilized with 0.5% Triton X-100, and subsequently blocked non-specific binding sites in phosphate buffered saline (PBS) containing 5% bovine serum albumin (BSA). The primary antibody was incubated at 4°C overnight, and the next day was incubated with fluorescently labeled secondary antibody for 1 hour at room temperature. After staining is complete, the nuclei were labeled with DAPI (Aladdin, China) and mounted with Fluoro-KEEPER Antifade Mounting Medium (Aladdin, China). Finally, images were observed and acquired by an Olympus IX53 fluorescence microscope (Olympus, Japan). Quantify at least 2 nuclei in at least 3 independent fields of view.

### 2.7 Histology

Cells were fixed with 4% paraformaldehyde (Beyotime, China) for 10 min to prepare 5 μm thick human lung tissue sections. Permeabilize with 0.5% Triton X-100 for 5 min, followed by blocking with 15% FBS for 1 h. Incubate the samples for 1 h with a 1:200 dilution of p-MYL12A (Abcam, USA) and a 1:100 dilution of MPO (Abcam, USA), both of which were diluted with 15% FBS. After 3 washes (5 min each) with TBST, incubate the samples with 1:800 dilution of goat anti-rabbit secondary antibody (Abcam, USA) in TBST for 45 min at room temperature. Nuclear staining was performed using 0.1 μg/mL DAPI (Thermo Fisher Scientific, USA).

### 2.8 Western blotting

Cell extracts were separated into detergent-soluble and detergent-insoluble fractions with 0.2% Triton X100 buffer [20 mM Tris-HCl (pH 7.4), 150 mM NaCl, 0.2% Triton-X100, 10% glycerol, and 1% protease inhibitor cocktail (Nacalai Tesque, Japan)]. Dissolve the detergent-insoluble fraction in 1% Triton X-100 buffer supplemented with 1% SDS and benzonase (Sigma, USA). The lysate (20 μg) was subjected to SDS-PAGE and transferred to a nitrocellulose membrane. Block the membrane with 5% BSA for 1 h at room temperature and incubate with the following primary antibodies: p-MYL12A rabbit monoclonal antibody (1:2000, Abcam, USA) and MYL12A rabbit monoclonal antibody (1:3000, Abcam, USA). Anti-rabbit IgG antibody (1:10,000, Abcam, USA) conjugated to HRP after washing the membrane with 1x TBST was incubated for 1 h at room temperature. Protein bands were visualized using an ECL detection system (GE Healthcare, USA) and band intensity was quantified using ImageJ software.

### 2.9 Obtain genes associated with LLPS

LLPS-related genes were downloaded from the PhaSepDB2.1 website (http://db.phasep.pro/) and only the genes encoding proteins were retained. This is an online database of all LLPS-related genes, with a total of 3215 genes downloaded for subsequent analysis.

### 2.10 Transcriptome data download and processing

The blood transcriptome and matching clinical data of 144 samples including patients with simple sepsis (sepsis), patients with sepsis + ARDS (sepsis), patients with SIRS and patients without sepsis, SIRS or ARDS (untreated) were downloaded from the GEO database item number (GSE32707). Further details regarding the entire cohort’s baseline characteristics and inclusion criteria can be found in the original publication associated with this dataset. Neutrophil transcriptome data from blood isolates from 23 ARDS patients and healthy controls were downloaded from item number (GSE76293). All transcriptome data were normalized using FPKM.

### 2.11 Single-cell sequencing data download and processing

In this paper, we downloaded single-cell data from GEO database item number (GSE157789), including 5 cases of sepsis ARDS and 5 healthy control groups of peripheral blood lymphocytes and leukocytes. We performed routine processing on single-cell data, including data normalization (NormalizeData), search for high-variable genes (FindVariableFeatures), data normalization (ScaleData), data dimensionality reduction (RunPCA), batch effect removal (RunHarmony), cell clustering (FindNeighbors, FindClusters), cell visualization (RunUMAP), Cell types were manually annotated based on the FindAllMarkers function and marker genes. All neutrophils were scored with LLPS score (GSVA) based on LCGs, and neutrophils were divided into high and low LLPS score groups using the median, and differential gene analysis (FindMarkers), gene enrichment analysis (GSEA) and cell communication analysis (Cellchat) were performed, all of which were performed in the R (4.4.0) environment.

### 2.12 Differential expression and enrichment analysis of mass spectrometry

In the mass spectrometry differential expression analysis, we preprocessed the data using unit variance scaling (UV) and used the Wilcoxon rank-sum test to identify differentially expressed proteins in ARDS patients versus healthy controls, independently calculated the p-value of each protein, and FDR corrected for the p-value using the Benjamini-Hochberg method. The screening criteria were the corrected p-value (FDR)<0.05 and |log2FC| > 1, which was finally determined to be differentially expressed proteins. Then, Kyoto Encyclopedia of Genes and Genomes (KEGG) enrichment analysis was performed on the differentially expressed proteins using the R-package clusterProfiler.

### 2.13 Construction of prognostic models

To explore the clinical prognostic value of the LLPS score and MYL12A, we performed a survival analysis on the cohort of 44 ARDS patients identified from the GSE32707 dataset. We quantified the LLPS enrichment score for each patient using the GSVA algorithm based on their peripheral blood transcriptome data. Patients were then dichotomized into high and low LLPS score groups based on the optimal cut-off point determined by the “maxstat.test” function.

### 2.14 Statistical analysis

The experimental data were expressed as SD ± mean, and the obtained continuous values were first tested for the homogeneity of variance, if the homogeneity of variance was satisfied, the T-test was used for the comparison of the mean of the two groups of independent samples, and the ANOVA variance test was used for the comparison of the mean of multiple groups of independent samples. GraphPad Prims 8 was used to plot the charts, and the data were analyzed using R, and the statistical test standard was *p*<0.05.

## 3 Result

### 3.1 ARDS neutrophils exhibit a unique LCGs expression profiles

**Figure 1:**
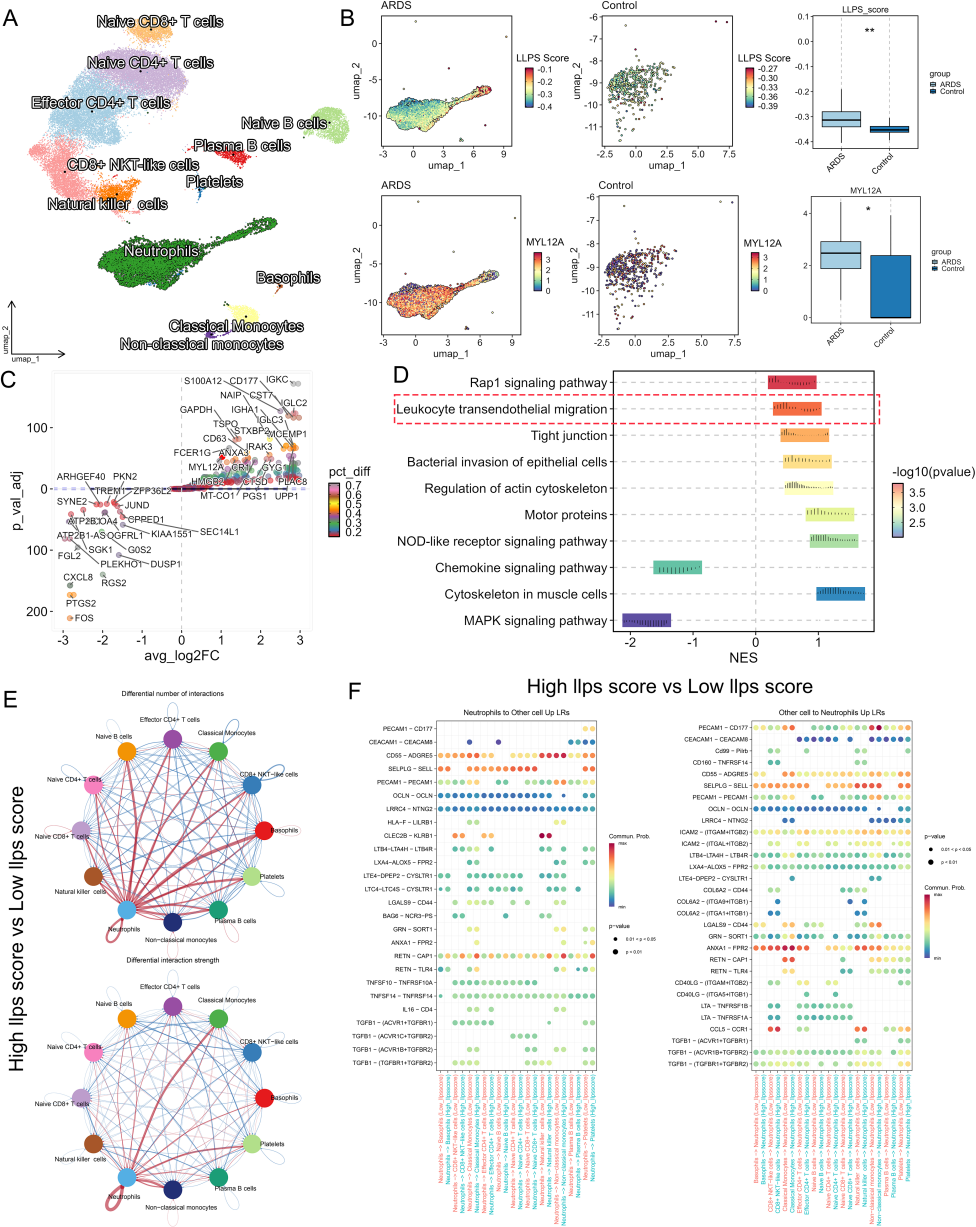
Single-cell transcriptomics identifies distinct neutrophil unique expression pattern in ARDS. **(A)** UMAP projection reveals the cell types of the ARDS peripheral blood, showing 12 clusters, each colored and representing a different cell line. **(B)** UMAP and bar plots demonstrate the GSVA scores of LLPS in ARDS versus healthy control groups. **(C)** Scatter plots reveal differential LLPS genes (x-axis: avg_log2FC; y-axis: p-val-adj; color intensity: percentage difference). Key upregulated genes (red) meet thresholds of |log2FC|>1 and FDR<0.05. **(D)** GSEA pathway analysis for LCGs [x-axis: normalized enrichment score (NES); y-axis: biological pathways; color intensity: -log10(p-value)]. Top enriched pathways related to neutrophil migration are labeled. **(E)** Cell-cell interaction networks demonstrating: (Top) quantitative changes in ligand-receptor pairs; (Bottom) interaction strength alterations (red: enhanced; blue: diminished in ARDS vs controls). **(F)** Compared to low LLPS scores, high scoring LLPS in dot plot highlighting significantly upregulated (red) ligandreceptor pairs. *p<0.05, **p<0.01.

Single-cell transcriptomic analysis of peripheral blood from 5 ARDS patients and 5 healthy controls identified 12 immune cell subsets (**Figure 1A**). GSVA scoring revealed significantly elevated LLPS activity in ARDS compared to controls (**Figure 1B**). Differential expression analysis identified >2-fold upregulation of key LCGs, including CD177 and MYL12A (**Figure 1C**). GSEA demonstrated significant enrichment of these genes in neutrophil chemotaxis and actin cytoskeleton remodeling pathways (**Figure 1D**).

CellChat analysis uncovered neutrophils in the high-LLPS cohort exhibited significantly enhanced ligand-receptor interaction intensity and pairing number compared to the low-LLPS group (**Figure 1E**). Notably, high-LLPS neutrophils demonstrated marked upregulation of ligand-receptor pairs functionally linked to transendothelial migration-including PECAM1-CD177, CD55-ADGRE5, and ANXA1-FPR2 interactions (**Figure 1F**). These findings demonstrate that sepsis-associated ARDS neutrophils acquire augmented polarization and migratory capacity through dysregulated expression of LLPS-related genes.

### 3.2 Multi-omics profiling identifies MYL12A as a key phase-segregated effector molecule for neutrophils in ARDS

**Figure 2:**
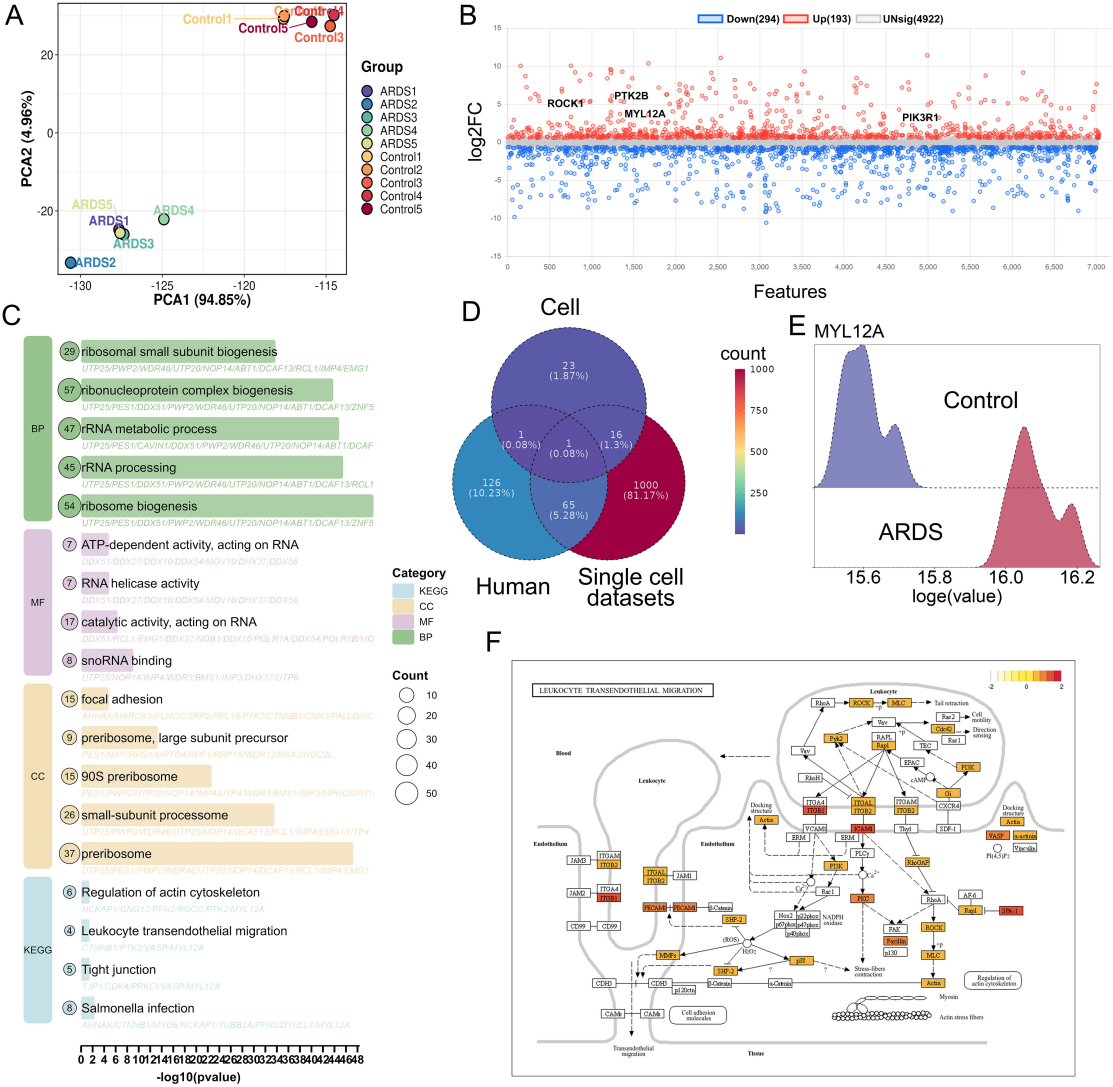
Identification of ARDS peripheral blood neutrophil protein profiling. **(A)** Principal component analysis demonstrates the proteins in the ARDS group were aggregated within the group and dispersed between groups, with good heterogeneity, x=differences between groups, and y=differences within groups. **(B)** The dot plot revealed that 193 proteins including ROCK1, PTK2B, MYL12A, and PIK3R1 were up-regulated and 294 down-regulated proteins in neutrophils of ARDS patients. Blue represents down-regulated genes, red represents up-regulated genes, and gray represents non-differential genes (|Log2FC|>1, *p-adj*<0.05|). **(C)** KEGG enrichment analysis of intersection genes showed that leukocyte transendothelial cell migration pathway-related genes were also enriched in immune inflammation-related pathways and natural killer cell-mediated cytotoxic pathways. **(D)** Venn diagram illustrating the multi-omics filtering strategy to identify the core LCG. The intersection reveals MYL12A as the only gene that is: 1) A known LLPS-associated gene (from the Human dataset, representing PhaSepDB); 2) Upregulated at the transcript level in ARDS neutrophils (from Single cell dataset); and 3) Identified at the protein level in phase-separated droplets from fMLP-stimulated neutrophils (from Cell dataset). **(E)** The ridge plot showed significantly higher expression of MYL12A in neutrophils in the ARDS group compared to the healthy control group. **(F)** KEGG database demonstrated that MYL12A is a gene in the RhoA/ROCK signaling pathway, which regulates neutrophil transendothelial cell migration.

In order to identify phase-separated genes associated with neutrophil migration, we extracted phase-separated droplets from neutrophils in fMLP-induced samples and from peripheral blood of ARDS patients. Principal component analysis (PCA) showed a significant difference in the protein profile characteristics of neutrophil phase separation droplets between the ARDS group and the healthy control group (**Figure 2A**). Proteomic analysis revealed that 193 proteins were significantly upregulated in neutrophil phase separation droplets of ARDS patients (**Figure 2B**). Interestingly, our proteomic analysis also revealed that proteins co-partitioning into these phase-separated droplets were significantly enriched in pathways associated with ribosome biogenesis and protein synthesis (**Figure 2C**). This suggests that MYL12A-mediated liquid-liquid phase separation may not only directly regulate the actin cytoskeleton to promote migration but also serve as a scaffold to organize and concentrate the translational machinery. Such a mechanism would effectively couple the cell’s migratory apparatus with its protein synthesis capacity, ensuring that neutrophils are fully equipped for their effector functions upon arrival at the site of inflammation. This highlights the potential role of liquid-liquid phase separation in coordinating multiple facets of neutrophil activation in ARDS. By cross-analyzing single-cell transcriptome, proteome, and fMLP stimulation datasets, MYL12A was identified as the only commonly differentially expressed LCG (**Figure 2D**), and MYL12A was significantly overexpressed in neutrophils of ARDS (**Figure 2E**). The KEGG database showed that MYL12A regulates cell tail retraction through phosphorylation activation, thereby promoting neutrophil migration (**Figure 2F**).

In summary, this study identified MYL12A as a key phase separation effector molecule for neutrophil migration in ARDS through multi-omics integrated analysis, and it plays a key role in ARDS by regulating cell polarization and migration.

### 3.3 LLPS score has clinical prognostic value, and a high LLPS score is associated with better overall survival

**Figure 3:**
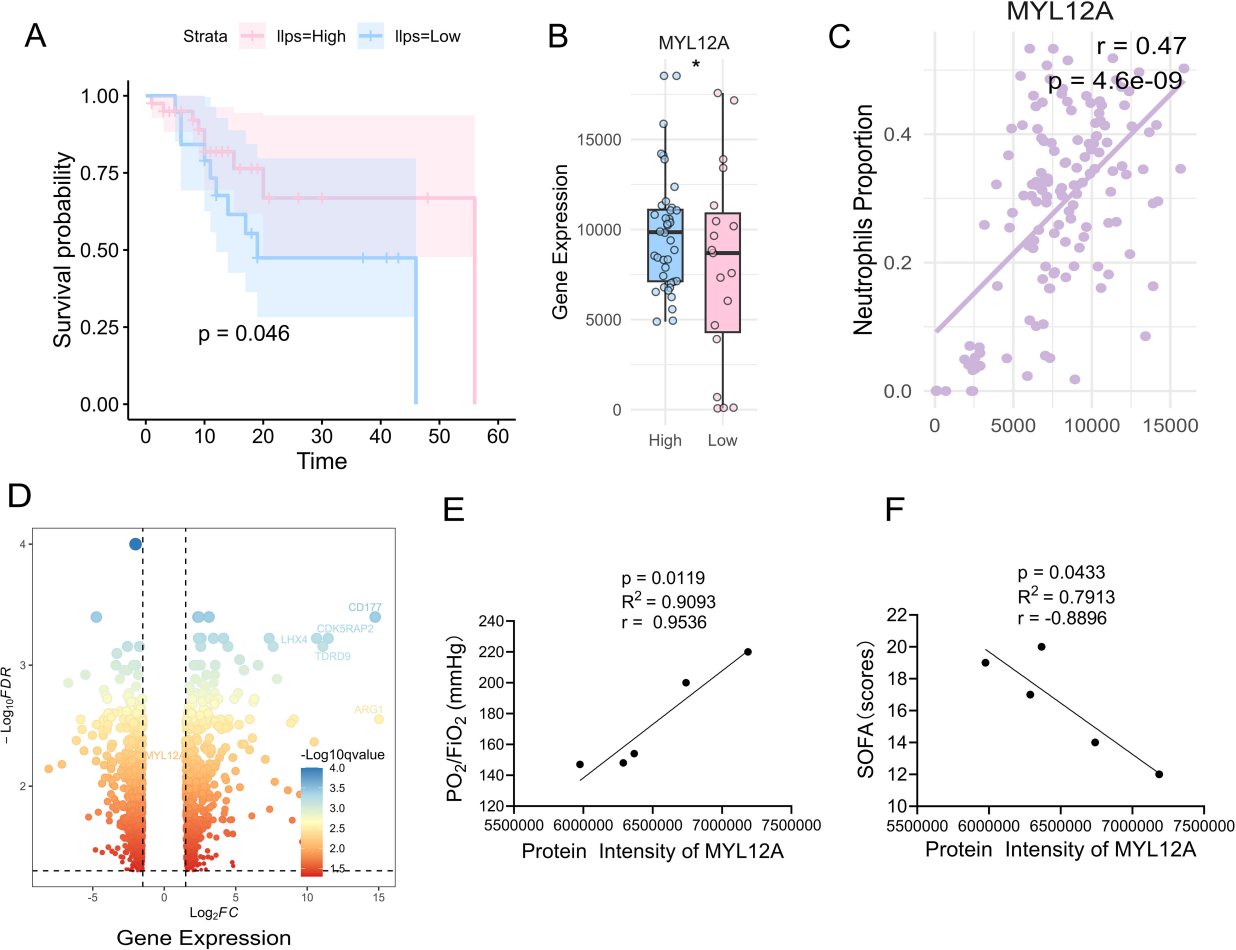
Correlation between LLPS score with LCGs and ARDS prognosis. **(A)** Kaplan-Meier curve analysis showed that patients with ARDS in the high LLPS group had a higher overall survival rate with lower ARDS in the LLPS group, *p* =0.046. **(B)** MYL12A in the high LLPS group was significantly up-regulated, *p*<0.05. **(C)** Spearman correlation analysis showed that the expression level of MYL12A gene was significantly positively correlated with the number of neutrophils, r=0.47, *p*<0.05. **(D)** Volcano plot showing high expression of MYL12A in ARDS with high LLPS scores. **(E)** Correlation analysis between phase separation droplet MYL12A concentration and oxygenation index in ARDS patients, r=0.9536, *p*=0.0119. **(F)** Correlation analysis between the concentration of MYL12A in phase separation droplets and the SOF score of ARDS patients, r=-0.8896, *p*=0.0433. *p<0.05.

In order to explore the clinical prognostic value of LLPS score and MYL12A in ARDS patients, this study quantified the LLPS enrichment score of individual patients by GSVA algorithm based on the peripheral blood leukome transcriptome data and clinical information of 44 ARDS patients included in the GEO database (see Methods), and divided the samples into high/low LLPS score groups according to the automatic segmentation point of prognostic analysis. Kaplan-Meier survival analysis showed that the overall survival rate (OS) of patients in the high LLPS score group was significantly better than that in the low LLPS group (*p*=0.046, **Figure 3A**), and the expression level of MYL12A in neutrophils in this group was significantly increased (**Figure 3B**). The correlation results showed a positive correlation with the proportion of neutrophils in peripheral blood (r=0.47, *p*<0.05, **Figure 3C**), which further supported the association with neutrophil function. In order to verify the source of transcriptome data MYL12A, we further analyzed an independent dataset of 23 ARDS patients and healthy controls, and found that the mRNA levels of MYL12A in peripheral blood neutrophils of ARDS patients were significantly upregulated (*p*<0.01, **Figure 3D**).

In addition, the clinical samples of 5 patients with ARDS were analyzed and found that the protein content of MYL12A in the droplets was significantly positively correlated with the oxygenation index of the patients (r=0.9536, *p*=0.0119, **Figure 3E**) and negatively correlated with the disease severity score (SOFA score) (r=-0.8896, *p*=0.0433, **Figure 3F**).

In summary, the overall survival rate of ARDS patients with high LLPS scores was significantly improved, suggesting that LLPS may be a key mechanism for the regulation of immune homeostasis. MYL12A in droplets may be used as a protective molecular marker for ARDS by maintaining neutrophil migration and functional balance: its elevated content is not only associated with the improvement of oxygenation function, but also significantly associated with the reduction of disease severity, providing a potential target for clinical prognosis evaluation and precision immune intervention.

### 3.4 p-MYL12A phase separation dynamically regulates cell migration

**Figure 4:**
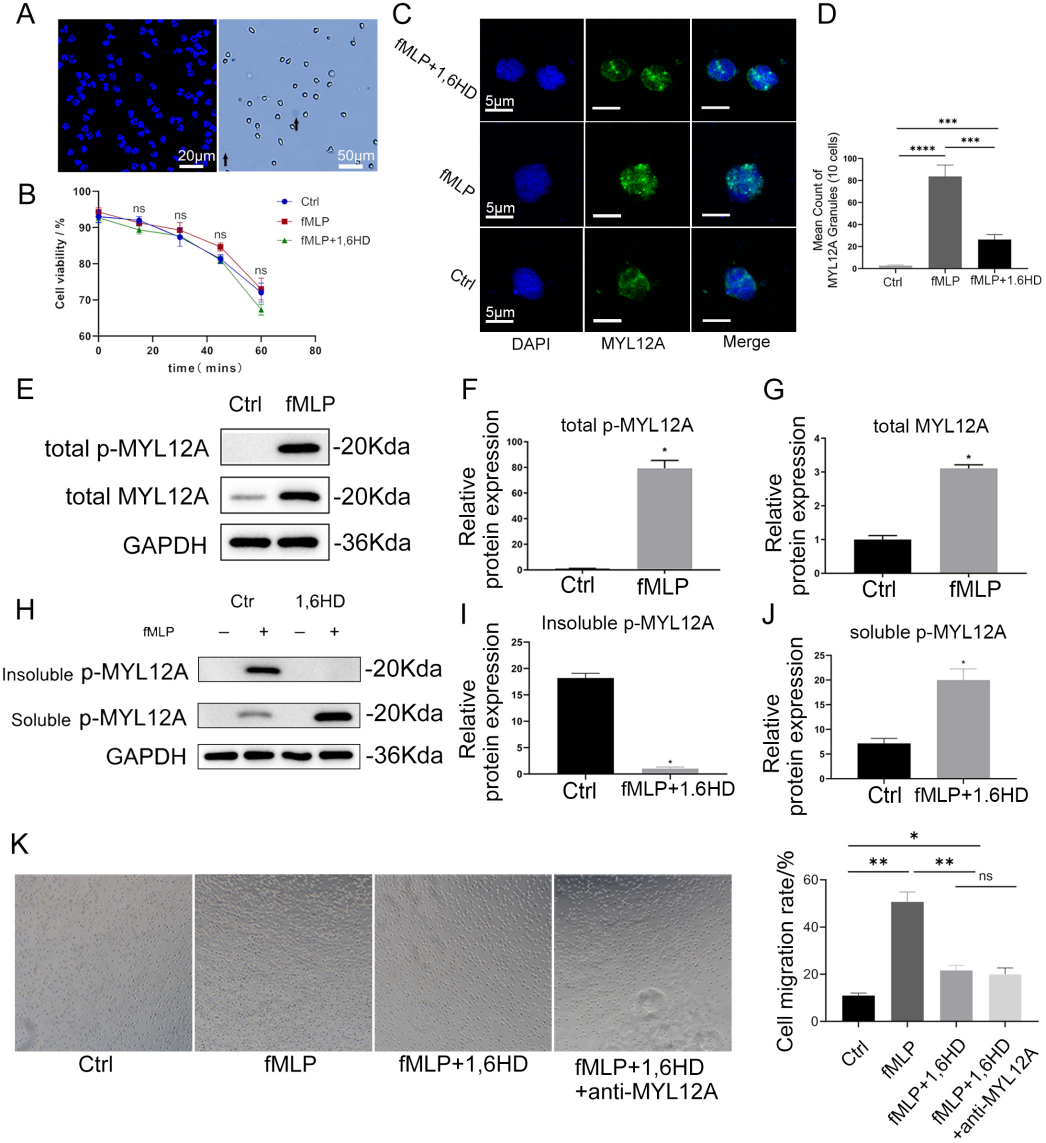
fMLP induces reversible phase separation of neutrophil p-MYL12A, which can be inhibited by 1, 6HD. **(A)** Left: After DAPI stained nuclei, neutrophil purity was calculated > 90% under fluorescence microscopy, bar=20 μm. Right: Trypan blue stained live cells are transparent and unstained, dead cells pointed by black arrows are stained blue, cell viability > 90%, bar=50 μm. **(B)** Neutrophil activity was detected every 15 min, and neutrophils were treated with 3% 1 and 6HD for 30 min. (n=3). **(C)** Confocal microscopy revealed the formation of MYL12A fluorescent spots after fMLP treatment with neutrophils, and the number of spots decreased after the addition of 3% 1,6-HD, bar=5 μm. **(D)** Fluorescent spots of 10 neutrophils were randomly counted, the number of spots increased significantly in the fMLP group and decreased after the addition of 1,6HD. (n=3). **(E)** Neutrophils were treated with fMLP and immunoblotted with specific antibodies. **(F, G)** fMLP induces an increase in p-MYL12A and MYL12A expression in neutrophils. (n=3). **(H)** Neutrophils were treated with fMLP (10 nM, 30 min), followed by 5 min with or without 3% 1,6-HD, cell extracts were extracted with Triton X-100, and immunoblotting was performed with specific antibodies. **(I)**. 1,6HD induces decrease of insoluble p-MYL12A in fluorescent spots. (n=3). **(J)**. 1,6HD induces significant increase of soluble p-MYL12A in fluorescent spots. (n=3). **(K)**. The number of migrating cells in the lower chamber of the Transwell after different treatments was observed in brightfield. (n=3). ns=no statistical difference, *p<0.05, **p<0.01, ***p<0.001, ****p<0.0001.

LLPS is driven by a network of weak, multivalent interactions, including hydrophobic, electrostatic, and π-π stacking forces (14, 15, 22). The aliphatic alcohol 1,6-HD is widely used to probe these interactions and disrupt the formation of liquid-like condensates. Isolated neutrophils demonstrated >90% purity and viability (**Figure 4A**), and treatment with 3% 1,6-HD for 30 minutes showed no significant cytotoxicity compared to control (Ctrl) or fMLP-stimulated groups (p>0.05, **Figure 4B**). Immunofluorescence revealed that fMLP stimulation (10 nM, 30 min) induced MYL12A to form fluorescent spots in the cytoplasm (**Figure 4C**). This fMLP-induced formation of MYL12A puncta was significantly reversed by treatment with 1,6-HD(p<0.001, **Figure 4D**), confirming that their assembly is predominantly driven by a process consistent with LLPS. We noted that 1,6-HD did not completely eliminate all puncta, which may suggest the presence of a heterogeneous population of condensates, with the residual structures potentially representing a more stable, gel-like state that is less sensitive to 1,6-HD.

Phosphorylation modulates phase separation capacity by altering protein surface charge states or conformations (23). Notably, the MYL12A phosphorylation site Ser19 resides within its predicted LLPS domain (residues 16-21), suggesting phosphorylation may directly regulate its phase separation state.

To validate phospho-MYL12A regulate neutrophils through reversible LLPS, we employed the *Takuya Noguchi* method (24). Triton X-100 separated the soluble and insoluble protein fractions, and found that p-MYL12A was significantly enriched in the insoluble fraction after fMLP stimulation (**Figures 4E-G**), while p-MYL12A was transferred from the insoluble to the soluble fraction after 1,6-HD treatment (**Figures 4H-J**), indicating that the phosphorylation state was closely related to the reversibility of phase separation. Transwell assays confirmed that fMLP significantly enhanced neutrophil migration (*p*<0.01), while pretreatment with 1,6-HD or anti-MYL12A antibody significantly inhibited this effect (**Figure 4K**).

Collectively, these results demonstrate that fMLP induces MYL12A-containing cytoplasmic condensates whose formation is inhibited by 1,6-HD, while transwell assays confirm that 1,6-HD blocks the fMLP-stimulated migration response, mechanistically linking MYL12A phase separation to neutrophil migration.

### 3.5 Neutrophils in ARDS lung tissue were highly expressed p-MYL12A

**Figure 5:**
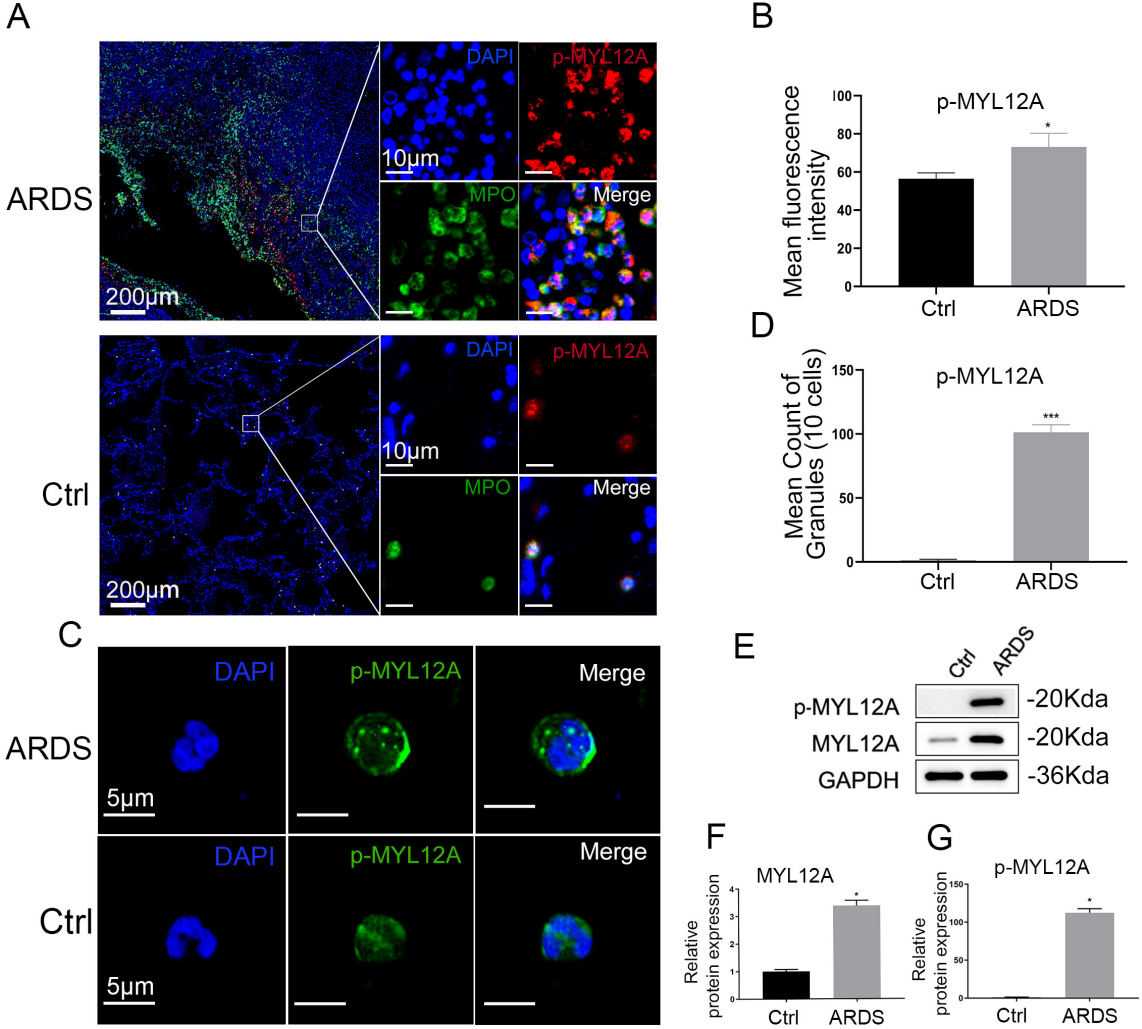
Polymerization formation of MYL12A in ARDS neutrophil **(A)** Neutrophils infiltrated abundantly in lung tissue of ARDS and high expression of p-MYL12A, blue=DAPI, red=MYL12A, green=MPO, left bar=200 μm, right bar=10 μm. **(B)** The mean fluorescence intensity of p-MYL12A in ARDS neutrophils was significantly increased. (n=3). **(C)** Neutrophil p-MYL12A formation of fluorescent spots in ARDS observed by fluorescence microscopy, bar=5 μm. **(D)** The number of fluorescent spots with 10 neutrophil counts p-MYL12A in the ARDS group was significantly higher than that in the control group. (n=3). **(E)** Neutrophils isolated from ARDS, immunoblotting with indicated antibodies after protein extraction. **(F, G)** Gray analysis of western blot bands showed that neutrophil expression of p-MYL12A and MYL12A was elevated in ARDS. (n=3). *p<0.05, ***p<0.001.

Immunofluorescence analysis showed that neutrophils in the lung tissues of patients with ARDS were significantly aggregated (**Figure 5A**), and their intracellular p-MYL12A expression levels were significantly higher than those in the healthy control group (**Figure 5B**). Further isolation of peripheral blood neutrophils for immunofluorescence detection showed that p-MYL12A showed dense cytoplasmic fluorescence spots in neutrophils of ARDS patients (**Figure 5C**), and random counting of the number of spots in 10 cells showed that the number of spots in the ARDS group was significantly higher than that in the control group *(p*<0.05, **Figure 5D**), indicating that the LLPS phenomenon was significantly enhanced.

Western blot validation showed that there was no significant difference in the total protein expression of MYL12A in neutrophils from ARDS compared with the healthy control group (**Figure 5E**), but the phosphorylation level of MYL12A was significantly increased (*p*<0.01, **Figures 5F-G**). Combined with immunofluorescence and Western blot, the results suggested that ARDS neutrophils drove LLPS through phosphorylation of p-MYL12A to form a phase-separated droplet structure, which in turn promoted their directional migration from peripheral blood to lung tissue.

## 4 Discussion

LLPS, as a fundamental molecular mechanism governing the formation of membraneless organelles, with growing evidence implicating its involvement in diverse physiological and pathological processes (25). In neurological diseases, aberrant LLPS has been extensively studied, and its role in tumors, viral infections and inflammatory responses increasingly gain attention. Under physiological conditions, LLPS participates in transcriptional regulation, genome stability maintenance, signal transduction, protein degradation, and DNA damage repair (26, 27). In ARDS, neutrophils undergo profound transcriptional and phenotypic reprogramming in response to inflammatory stimuli and microenvironmental changes (20).

In this study, through multi-omics integrated analysis and functional verification, the regulatory role of LLPS in ARDS neutrophils was systematically revealed, and ARDS neutrophils showed unique LLPS-related genes (LCGs) expression characteristics. MYL12A drives reversible phase separation by phosphorylation of Ser19, dynamically regulating neutrophil migration. High LLPS activity was significantly associated with a good prognosis. These findings provide a new perspective for understanding the immune regulation mechanism of ARDS. From the perspective of molecular mechanism, this study found that MYL12A-mediated phase separation has the following characteristics: firstly, bioinformatics prediction showed that the N-terminus of MYL12A contains intrinsically disordered regions (IDRs) and phosphorylation regulatory modules, which provides a structural basis for its phase separation ability. =More importantly, the reversible changes of p-MYL12A fluorescent spots were found when the soluble and insoluble fractions of Triton X-100 were extracted from the cells treated with 1, 6HD, respectively. These findings echo recent research, but for the first time reveal this regulatory pattern in neutrophils. In terms of clinical significance, this study has important findings. First, the LLPS scoring system showed good prognostic value, and the 28-day survival rate of patients in the high-scoring group was significantly improved (68.2% vs 40.9%). Secondly, the phase separation level of MYL12A was significantly correlated with disease severity.

The pathological role of neutrophils in ARDS has been extensively investigated. Conventional paradigms posit that inhibiting neutrophil chemotaxis, migration and reducing neutrophil-mediated oxidative stress improve ARDS outcomes (28). A critical point raised by these findings is how to reconcile the protective nature of enhanced neutrophil migration with its well-established role in causing tissue damage. Our study suggests that MYL12A-mediated LLPS does not simply amplify migration, but rather acts as a sophisticated regulatory hub. This mechanism appears to function as a ‘molecular switch’, enabling neutrophils to transition between pro-inflammatory and anti-inflammatory states, thereby preventing uncontrolled tissue infiltration and the subsequent inflammatory cascade. By maintaining the homeostasis of actin remodeling, LLPS ensures that migration is directional and precise, which is crucial for an effective immune response without excessive collateral damage. This regulatory role, rather than simple enhancement of migration, likely explains the observed positive correlation between high LLPS scores and improved patient survival. This study identifies reversible phase separation of phosphorylation-MYL12A as a protective mechanism—consistent with some of the known findings. For example, LLPS enables immune cells respond quickly to environmental stimuli under stressful conditions by forming reversible biomolecular condensations such as stress granules (29). The formation of SGs sequesters key RNAs and proteins, preventing loss of function due to oxidative damage or heat shock (30–32). Similarly, the dynamic phase separation of MYL12A may play a protective role through the following mechanisms: its phase separation properties endow the RhoA/ROCK pathway with a “molecular switch” function, enabling neutrophil pro-inflammatory and anti-inflammatory types to switch to each other, thereby inhibiting over-activated neutrophil migration and avoiding the loss of control of the lung inflammatory cascade; Secondly, by maintaining the homeostasis of actin remodeling, the directional migration and functional performance of neutrophils at the site of inflammation are ensured. This dynamic regulatory model is highly similar to the role of HIP-55 in heart failure. HIP-55 inhibits the overactivation of the p38/MAPK pathway by sequestering β-adrenergic receptors through phase separation (33). This finding suggests that LLPS is not only a participant in the inflammatory response, but also a key regulator of immune homeostasis, reflecting its important role in maintaining immune homeostasis.

Despite these important advances, our study has limitations. First, as rightly pointed out, some of the clinical correlation analyses were performed with a small sample size. While statistically significant, these findings should be interpreted as preliminary, and their robustness needs to be confirmed in future studies with larger, independent patient cohorts. Second, the transient viability of ex vivo neutrophil cultures precludes longitudinal tracking of phase separation dynamics in real time. Definitive *in vivo* proof would require further experimental approaches, such as the use of knock-in mice with mutations at the Ser19 phosphorylation site. Also, this study did not directly examine whether these condensates contain RNA or classical stress granule marker proteins. Therefore, elucidating the complete molecular composition of MYL12A condensates and their relationship with other known membraneless organelles, such as stress granules, will be an important direction for our future research. Finally, although the phase separation properties of MYL12A are related to its protective effect, the interaction network between MYL12A and other phase-separated proteins remains unclear, limiting a full understanding of the condensates’ composition and function.

Additionally, while our multi-omics data strongly point to a link between the enriched RhoA/ROCK pathway and MYL12A function, we did not experimentally validate the direct impact of this pathway on the formation of MYL12A condensates, for instance, by using specific RhoA/ROCK inhibitors. Such experiments are challenging due to the short *ex vivo* lifespan of primary neutrophils. Therefore, future studies, potentially using more stable cellular systems, are required to definitively establish this direct mechanistic causality and to further dissect the upstream signaling architecture governing MYL12A phase separation in ARDS.

Collectively, this study not only reveals the central role of LLPS in the regulation of neutrophil function, but also opens up a new strategy for the treatment of “targeted phase separation”, which provides a new strategy for the diagnosis and treatment of ARDS. With the deepening of research, phase separation regulation is expected to become a new target for the treatment of inflammatory diseases, advancing precision medicine paradigms.

## Data Availability

The datasets presented in this study can be found in online repositories. The names of the repository/repositories and accession number(s) can be found in the article/**Supplementary Material**.
